# **Dysphagia and its impact on the quality of life of head and neck cancer patients: institution-based cross-sectional s**tudy

**DOI:** 10.1186/s13104-020-05440-4

**Published:** 2021-01-07

**Authors:** Tseganesh Asefa Yifru, Sezer Kisa, Negalign Getahun Dinegde, Niguse Tadele Atnafu

**Affiliations:** 1 School of Nursing, College of Medicine and Health Sciences, University of Gondar, Gondar, Ethiopia; 2 Department of Nursing and Health Promotion, Faculty of Health Sciences, Oslo Metropolitan University, Oslo, Norway; 3 Department of Nursing, School of Nursing & Midwifery, College of Health Sciences, Addis Ababa University, Addis Ababa, Ethiopia; 4 Department of Nursing, School of Nursing & Midwifery, College of Health Sciences, Addis Ababa University, Addis Ababa, Ethiopia

**Keywords:** Dysphagia, Head and neck cancer, Impaired swallowing, MDADI, Quality of life

## Abstract

**Objective:**

Impaired swallowing is a primary medical concern in head and neck cancer (HNC) patients**.** Swallowing therapy and supportive care to relieve swallowing problems among HNC patients are recommended. However, no data shows the effect of dysphagia on the quality of life (QoL) among Ethiopian patients. This cross-sectional study aimed to assess swallowing function and its impact on QoL.

**Results:**

The sample included 102 HNC patients who visited oncology clinics at Tikur Anbessa Specialized Hospital. Majority were male (53.90%), employed (70.6%), single (57.80%), and completed some level of formal education (66.60%) with a mean age of 42.58 years (SD ± 14.08). More than half of the patients (69.6%) medical expenses were covered by the government. Most were suffering from advanced stage HNC (59.80%), squamous cell carcinoma (62.70%), and the most prevalent tumor location was nasopharynx (40.20%). The mean MDADI score was 53.29 (SD ± 15.85). Being female, low income, suffering from laryngeal cancer, advanced tumor, and undergoing a single modality therapy were crucial determinants of poor QoL related to swallowing problems. It is recommended to assess swallowing related QoL of patients using a validated tool and be included in treatment protocols.

## Introduction

Dysphagia is a frequent sequel of HNC and its treatment. Patients report significant difficulty eating solid foods, swallowing, choking**,** coughing after the swallow, spending more time consuming food and weight loss which exacerbate with treatment intensification. HNC also has a profound impact on the social well-being of patients, such as altered social interactions, loss of enjoyment with eating, embarrassment, and dissatisfaction throughout treatment and even after completion of a therapy [1–3].

Factors predictive of poor QoL due to dysphagia include patient-related factors such as smoking, age and gender [1, 4–9]; tumor-related factors such as advanced tumor stage [10–12] and factors related to tumor location [11, 13]. It is well established in the literature that patients with tongue [13, 14], buccal [12] and hypopharyngeal [10, 15, 16] tumors had worse dysphagia related QoL. Patients who have had radiotherapy treatment [6, 17–19], HNC surgery [20], and concomitant chemotherapy [13] had higher levels of dysphagia and dietary problems that indicate lower QoL. Deterioration in swallowing function affects the patient’s QoL multidimensional throughout the course of a treatment [21].

Studies highlight the need for assessing the QoL of cancer patients for a good understanding of the degree of improvement obtained with therapeutic procedures. Despite this fact, no study in Ethiopia assessed this association, and therefore, this study analyzed the association between dysphagia and QoL among patients undergoing treatment for HNC in Ethiopia.

### Main text

#### Methods

##### Patients and sampling strategy

This cross-sectional study population included male and female adult head and neck cancer survivors who visited oncology clinics at Tikur Anbessa Specialized Hospital from February to April 2019. Patients who are ≥ 18 years, had no evident cognitive impairment and an eating disorder were selected using a consecutive sampling method and included in the study.

The sample size was estimated using the population adjustment formula for single proportion estimation based on a 95% confidence level, a precision of 5%, and 50% expected prevalence. Since there was no prior study and a 10% non-response, yielding a total sample of 109 head and neck cancer patients.

#### Data collection tools

##### Document review

Patient charts were reviewed to identify patients with HNC and extract clinical characteristics such as diagnosis, primary cancer site, cancer stage, type of carcinoma, and treatment type.

#### Self-reported questionnaire

A self-reported questionnaire was also used to collect information on patients' socio-demographics such as age, sex, marital status, level of education, working condition, residence, income, and modality of payment coverage for medical expenses.

#### The MD Anderson Dysphagia Inventory (MDADI)

An Amharic version of the MDADI questionnaire (20-item psychometrically validated and reliable) is specifically developed to evaluate dysphagia`s impact on the QoL of patients with HNC [22], was used. All questions (except E7 and F2) has five possible responses (strongly agree, agree, no opinion, disagree, and strongly disagree) and scores from 1 (strongly agree) to 5 (strongly disagree). The Global domain is shown separately. The composite mean score is obtained by adding the other three domains, calculating their mean, and multiplying this by 20. The composite mean score ranged from 20 (extremely low functioning) to 100 (high functioning).

### Statistical analysis

This measure's overall reliability was determined to be very good (Cronbach's coefficient alpha = 0.924), and the subscale also had acceptable internal consistency (emotional, 0.79; functional, 0.71; physical, 0.89).

Questionnaire responses were used to construct categorical variables. Patients diagnosed with cancer within six months before data collection were defined as `newly diagnosed,` and patients who were divorced, widowed, separated, and never married are categorized as `single.` The level of income was determined based on their ability to cover their medical expenses. Patients were classified as `fee-paying` if they were economically able to pay their medical expenses, and those involved in any income-generating activity were categorized as employed. Furthermore, patients who took any combination of treatment modalities were categorized under 'Multi-modality treatment,' and cancer stages III & IV were categorized under 'Advanced.'

Descriptive statistics were used to analyze the demographic and clinical characteristics of participants. Besides, the independent t-test (for two groups) and one-way ANOVA (for more than two groups) were used to compare means between the demographic and clinical features with four domains, and the composite mean score of the MDADI and *P* < 0.05 was considered significant. Once it was determined that differences exist among the means, post hoc range tests and multiple pairwise comparisons were conducted to determine which means differ. All statistical tests were performed using version 24 SPSS (Statistical Package for Social Sciences).

### Results

#### Patient characteristics

The study population consisted of 102 HNC patients (93.6% response rate), mostly males (53.90%), employed (70.6%), single (57.80%), and completed some level of formal education (66.60%) with a mean age of 42.58 years (SD ± 14.08 years). The majority (69.6%) belonged to extremely low economic status, and the government covered their medical expense. Most were suffering from advanced stage HNC (59.80%), and squamous cell carcinoma 64 (62.70%), and the prevalent tumor location was nasal cavity/ nasopharynx carcinoma (40.20%), and about 34.3% of patients were newly diagnosed. Combination of treatment modalities, including chemotherapy, radiation therapy, and surgery, was the most prevalent type of treatment in which 55.90% of the patients had this all treatments (Additional file 1: Tables S1 and S2).

#### MDADI scores

The composite mean MDADI score of 53.29 (15.85) and overall question score of 53.34 (16.96) was found, and the lowest scores were found for the global score (44.51) and physical domain (49.4) (Additional file 1: Tables S3).

Tables 1 and 2 shows the mean differences in MDADI scores among different groups. Among socio-demographic characteristics, women had a significantly lower composite mean (*p* = 0.036) and functional (*p* = 0.030) scale scores. Patients who were economically able to pay for their medical expenses had significantly higher composite mean scores in all MDADI domains except the global domain. Other socio-demographic variables, including age, marital status, level of education, employment status, residence, and smoking habit, did not differ between each domain (Table 1).

**Table 1:** Mean differences of MDADI subscales according to socio demographic characteristics of participants

Variable	MDADI (Mean ± SD)						
	All questions	Composite mean	Global	Emotional	Functional	Physical	
Sex							
Male	56.3 ± 18.5	56.3 ± 16.6	47.6 ± 25.1	58.7 ± 18.9	61.2 ± 17.6		52.6 ± 21.3
Female	49.8 ± 14.4	49.7 ± 14.3	40.8 ± 19.9	54.3 ± 15.3	53.6 ± 16.8		45.7 ± 15.9
*P*	0.054	0.036	0.139	0.204	0.030		0.069
Age in years							
18–43	50.7 ± 16.0	51.6 ± 14.9	41.8 ± 22.6	53.7 ± 17.1	55.3 ± 17.7	47.1 ± 17.8	
Above 43	56.5 ± 17.7	55.3 ± 16.8	47.8 ± 23.3	60.2 ± 17.3	60.5 ± 17.0	52.3 ± 20.7	
* P*	0.086	0.241	0.189	0.060	0.140	0.169	
Marital status							
Living alone	55.4 ± 16.8	54.7 ± 15.5	47.8 ± 24.4	58.9 ± 17.3	59.3 ± 16.8	51.5 ± 19.1	
Living with partner	50.4 ± 16.9	51.4 ± 16.3	40.0 ± 20.5	53.6 ± 17.4	55.5 ± 18.5	46.6 ± 19.4	
* P*	0.141	0.301	0.091	0.130	0.292	0.210	
Level of education							
No formal education	52.7 ± 14.7	53.3 ± 13.6	44.7 ± 20.3	55.2 ± 15.6	56.0 ± 15.0	49.8 ± 16.8	
Primary	50.6 ± 14.8	50.4 ± 13.9	39.2 ± 20.8	53.9 ± 15.8	53.2 ± 17.0	48.0 ± 16.5	
Secondary	54.1 ± 22.5	52.9 ± 21.2	50.0 ± 28.5	57.2 ± 21.5	60.7 ± 20.1	48.1 ± 25.7	
Higher	56.2 ± 17.8	56.3 ± 16.5	45.4 ± 24.4	60.6 ± 18.3	62.0 ± 18.8	51.3 ± 20.5	
* P*	0.705	0.630	0.508	0.534	0.263	0.929	
Employment status							
Employed^a^	54.8 ± 17.6	54.4 ± 16.6	46.9 ± 24.5	58.0 ± 17.9	59.3 ± 17.6	50.8 ± 20.4	
Unemployed	49.9 ± 14.9	50.6 ± 13.7	38.7 ± 18.1	53.3 ± 15.9	53.9 ± 17.1	46.3 ± 16.1	
* P*	0.187	0.263	0.098	0.219	0.157	0.283	
Place of residence							
Addis Ababa	48.8 ± 17.1	49.1 ± 17.7	39.2 ± 22.9	52.9 ± 17.5	55.1 ± 19.1	43.8 ± 20.3	
Regional /rural	54.9 ± 16.7	54.7 ± 15.0	46.3 ± 22.9	57.9 ± 17.4	58.6 ± 17.0	51.4 ± 18.6	
* P*	0.118	0.119	0.177	0.214	0.382	0.081	
Level of income/medical expenses coverage							
Fee paying	60.7 ± 19.7	58.9 ± 19.8	50.9 ± 25.7	64.3 ± 17.9	65.7 ± 19.3	56.8 ± 24.2	
Covered by government	50.1 ± 14.6	50.8 ± 13.2	41.7 ± 21.3	53.3 ± 16.2	54.2 ± 15.6	46.2 ± 15.8	
* P*	0.003*	0.018*	0.061	0.003*	0.002*	0.010*	
Smoking							
Yes	52.3 ± 15.4	52.4 ± 14.9	43.4 ± 22.2	56.4 ± 15.6	56.6 ± 16.6	47.9 ± 17.8	
No	57.5 ± 22.3	56.8 ± 19.5	49.0 ± 26.3	57.5 ± 24.1	62.0 ± 21.0	55.6 ± 24.0	
* P*	0.229	0.266	0.333	0.806	0.222	0.109	

*t* test (for two groups comparison) and one-way ANOVA (for three and above group comparison) were employed, *Significant at *p* value less than 0.05

**Table 2:** Mean differences of MDADI subscales according to clinical and treatment variables of participants

Variable		MDADI (Mean ± SD)				
	All questions	Composite mean	Global	Emotional	Functional	Physical
Time since diagnosis						
New	52.1 ± 17.1	51.9 ± 15.4	44.6 ± 24.3	55.9 ± 16.9	56.5 ± 17.9	47.4 ± 19.0
6–12 months	52.9 ± 17.4	53.1 ± 16.2	46.7 ± 24.4	54.4 ± 18.9	55.8 ± 17.2	50.7 ± 18.9
More than year ago	55.3 ± 16.6	55.1 ± 16.3	41.9 ± 20.2	60.1 ± 16.2	61.3 ± 17.4	50.2 ± 20.3
P	0.729	0.333	0.707	0.389	0.389	0.749
Primary tumor site						
Oral cavity/Oropharyngeal	57.4 ± 20.8	57.4 ± 18.4	52.0 ± 28.1	59.3 ± 20.9	60.7 ± 20.5	54.5 ± 22.8
Nasal cavity/Nasopharyngeal	55.0 ± 16.4	54.7 ± 16.1	43.9 ± 21.1	59.0 ± 16.4	59.9 ± 16.9	50.8 ± 19.8
Larynx/ Hypo pharyngeal	47.2 ± 11.5	47.5 ± 11.2	38.1 ± 18.2	50.9 ± 13.8	51.9 ± 14.1	42.7 ± 12.2
*P*	0.044*	0.040*	0.058*	0.086	0.084	0.045*
Tumor T stage						
Initial	57.5 ± 18.2	57.7 ± 15.8	50.7 ± 24.9	60.5 ± 18.4	61.8 ± 16.8	53.4 ± 20.8
Advanced	50.6 ± 15.6	50.3 ± 15.3	40.3 ± 20.8	54.0 ± 16.4	54.9 ± 17.6	46.8 ± 17.9
*P*	0.042*	0.019*	0.024*	0.067	0.049*	0.092
Type of carcinoma						
Squamous cell	53.0 ± 16.6	53.3 ± 15.8	44.4 ± 22.1	56.0 ± 17.8	57.2 ± 17.2	49.5 ± 18.8
Adenocarcinoma	55.6 ± 19.5	54.7 ± 17.4	48.7 ± 25.5	58.3 ± 18.9	60.2 ± 20.2	51.5 ± 22.0
Other	51.3 ± 15.1	51.2 ± 14.4	38.7 ± 23.3	56.7 ± 13.9	56.0 ± 15.0	45.8 ± 17.2
*P*	0.730	0.803	0.425	0.874	0.725	0.676
Treatment modality						
Single modality treatment	50.5 ± 14.7	50.1 ± 14.0	40.9 ± 20.7	55.4 ± 15.9	55.7 ± 16.9	45.2 ± 16.7
Multi-modality treatment	55.5 ± 18.3	55.8 ± 16.9	47.2 ± 24.5	57.5 ± 18.6	59.2 ± 17.9	52.6 ± 20.6
*P*	0.148	0.072	0.170	0.555	0.328	0.054

*t*-test (for two groups comparison) and one-way ANOVA (for three and above group comparison) were employed, *Significant at *p* value less than 0.05

Regarding clinical features, statistically significant mean differences were observed between different primary tumor sites in all domains except the emotional and functional domains. In addition, patients with initial tumor stages (TI and II) had better scores in total questions (*p* = 0.042), composite mean score (*p* = 0.019), global (*p* = 0.024). and functional (*p* = 0.049) domains, while worse scores in the physical domain (*p* = 0.054) were observed among patients undergoing single modality treatment. Time since diagnosis and type of carcinoma did not differ between each domain (Table 2).

Compared with patients with oral cavity/ oropharyngeal cancer, those patients with laryngeal/ hypo pharyngeal cancer had significantly lower score in total questions (*p* = 0.048), composite mean score (*p* = 0.039), global (*p* = 0.047) and physical (*p* = 0.042) domains (Table 3).

**Table 3:** Multiple comparisons (Tukey HSD) between primary tumor site

Dependent variable	Primary tumor site	Primary tumor site	Mean difference	*P*	95% CI	
					Lower	Upper
Total questions	Oral cavity	Nasal cavity	2.3	.830	− 7.2	11.8
		Larynx	10.2^*^	.048	0.1	20.3
	Nasal cavity	Larynx	7.9	.120	− 1.6	17.3
Composite mean	Oral cavity	Nasal cavity	2.7	.748	− 6.2	11.6
		Larynx	9.8^*^	.039	0.4	19.3
	Nasal cavity	Larynx	7.1	.135	− 1.7	15.9
Global	Oral cavity	Nasal cavity	8.1	.299	− 4.8	21.0
		Larynx	13.9*	.047	0.2	27.7
	Nasal cavity	Larynx	5.8	.525	− 7.0	18.6
Physical	Oral cavity	Nasal cavity	3.7	.701	− 7.1	14.4
		Larynx	11.8^*^	.042	0.4	23.3
	Nasal cavity	Larynx	8.2	.167	− 2.5	18.9

^*^The mean difference is significant at the 0.05 level

### Discussion

This study aimed to assess dysphagia's impact on the quality of life domains of the MD Anderson Dysphagia Inventory among 102 head and neck cancer patients. We found a composite mean of 53.29 in the MDADI, reflecting a mean reduction in the QoL due to swallowing changes. The majority of patients in this study reported that their swallowing limits their daily activities.

The composite MDADI mean scores in this study were considerably lower than reported in Brazil (63.36) [3, 23, 24], the USA (64.06 and 67) [10, 23, 24], and Taiwan (67.08) [25], indicating the severity of this problem in Ethiopia. The variation may be due to the fact that patients in Ethiopia lack access to early treatment and support since there is a single cancer center in the country that provides comprehensive cancer care.

Similar to previous studies, factors such as gender [6], financial burden [26], tumor stage [27], location of HNC [18], and type of treatment [13, 14], this study also identified female sex, low income, suffering from hypopharyngeal cancer, and advanced tumor as the critical determinants of poor QoL related to swallowing problems among HNC patients. These results have direct implications for improving the care of HNC patients by highlighting the need to incorporate swallowing function in the assessment and clinical management of patients.

In contrast to previous studies that reported factors such as age [11, 17, 28], marital status [15], smoking [6, 18–20], and deterioration in swallowing function through time [12], no associations were observed between age, smoking habit, marital status, time since diagnosis and type of carcinoma in this study.

Female cancer patients in this study experienced poor swallowing related QoL consistent with earlier studies [11, 13]. Women were more emotionally distressed, frustrated, embarrassed, and depressed due to the inability to enjoy mealtime [29] compared with their male counterparts.

In this study, low-income patients scored lower MDADI scores in most of the domains, consistent with the previous studies that linked financial burden from cancer with poor QoL [10, 11].

Our results were consistent with prior studies that identified the negative impact of cancer stage and location on swallowing related QoL. It is interesting to note that worse MDADI scores among patients with advanced tumor stages and had hypopharyngeal tumors observed in prior studies [10, 23, 24]. The literature results were consistent with the risk observed for lower QoL scores in our population. Patients with oral cavity/ oropharyngeal tumor and at initial tumor stages had better MDADI scores than patients with hypopharyngeal and advanced-stage cancer, emphasizing the need to rigorously address swallowing function in such cancer patients.

Unlike the finding of studies done in Spain, Brazil, and the USA [13, 14], those patients who received a multi-modality treatment were found to have better scores than those with single-modality treatment. This difference might be related to the fact that those patients with advanced stage cancer were less likely to had surgery and radiotherapy, and any form of combination therapy due to their condition.

### Conclusion

In this cross-sectional study, patients with head and neck cancer had an impaired swallowing related quality of life. This study identifies factors associated with impaired swallowing related quality of life, such as gender (male), marital status (living alone), patient residence distance from the treatment center, and medical expense.

Therefore, future studies should separately address therapy's effect on swallowing problems for patients with early and advanced stage HNC patients in Ethiopia. As the burden of swallowing difficulties in HNC patients was established, it is crucial to incorporate swallowing function in routine cancer therapy protocols to mitigate dysphagia's effect on QoL.

### Strengths and limitations

To our knowledge, this study was the first in Ethiopia that assessed the effect of dysphagia on QoL and identified associated factors for poor dysphagia related QoL in HNC patients. It provided baseline data that are essential to evaluate the progress of swallowing function in HNC patients. However, some limitations of this study warrant caution in interpreting the results. Mainly, the cross-sectional nature of this study does not allow drawing any causal inferences. However, owing to the absence of literature in this area, our results could direct future studies by allowing hypothesis generation and providing useful information to target vulnerable populations. As self-reporting was used to collect patient data, our study was prone to recall bias. Furthermore, our questionnaire tailored to account for Ethiopian patients' cultural sensitivities may not be suitable for broader adoption without further validation.

## Supplementary Information

**Additional file 1: Table S1.** Socio Demographic Characteristics of Participants. **Table S2.** Clinical Characteristics of Participants**. Table S3.** Mean and Standard Deviation of MDADI.

## Data Availability

The datasets used in this study are available upon request.

## Abbreviations

ANOVA: Analysis of Variance
ETB: Ethiopian Birr
HNC: Head and neck cancer
MDADI: MD Anderson Dysphagia inventory
QoL: Quality of Life, SD: Standard Deviations
SPSS: Statistical Package for the Social Sciences
USA: United States of America
