# Supplementary material for: Dysphagia and its impact on the quality of life of head and neck cancer patients: institution-based cross-sectional study
Source: BMC Res Notes. 2021 Jan 7;14:11. doi: 10.1186/s13104-020-05440-4 (PMC7791825; doi:10.1186/s13104-020-05440-4)
Supplement: Supplementary file 1 — Additional file 1: Table S1. Socio Demographic Characteristics of Participants. Table S2. Clinical Characteristics of Participants. Table S3. Mean and Standard Deviation of MDADI. [file 13104_2020_5440_MOESM1_ESM.docx]

**Table S1. Socio Demographic Characteristics of Participants**

| **Variable** | | **Frequency** | **%** |
| --- | --- | --- | --- |
| **Sex** | Male | 55 | 53.90 |
|  | Female | 47 | 46.10 |
| **Age (Mean ± SD => 42.58±14.075)** | 18 to 43 years | 56 | 54.90 |
|  | Above 43 years | 46 | 45.10 |
| **Marital Status** | Living alone | 59 | 57.80 |
|  | Living with partner | 43 | 42.20 |
| **Level of Education** | No formal education | 34 | 33.30 |
|  | Primary school | 24 | 23.50 |
|  | Secondary school | 18 | 17.60 |
|  | Above secondary | 26 | 25.50 |
| **Working Condition** | Employed | 72 | 70.60 |
|  | Unemployed | 30 | 29.40 |
| **Place of Residence** | Addis Ababa | 26 | 25.50 |
|  | Regional/Rural | 76 | 74.50 |
| **Medical Expenses coverage** | Self | 31 | 30.40 |
|  | Government | 71 | 69.60 |
| **Smoking** | Not at all | 82 | 80.4 |
|  | Yes | 20 | 19.6 |

**Notes:** SD: Standard Deviations

**Table S2. Clinical Characteristics of Participants**

| **Variable** | | **Frequency** | **%** |
| --- | --- | --- | --- |
| **Time since diagnosis** | Less than 6 months | 35 | 34.3 |
|  | 6 to 12 months | 36 | 35.3 |
|  | More than a year ago | 31 | 30.4 |
| **Primary tumor site** | Oral cavity/Oropharyngeal | 30 | 29.4 |
|  | Nasal cavity/ Nasopharyngeal | 41 | 40.2 |
|  | Larynx/ Hypo pharyngeal | 31 | 30.4 |
| **Tumor T stages** | Initial (T1 & T2) | 41 | 40.2 |
|  | Advanced T3 & T4 | 61 | 59.8 |
| **Type of carcinoma** | Squamous Cell carcinoma | 64 | 62.7 |
|  | Adenocarcinoma Cell | 23 | 22.5 |
|  | Other | 15 | 14.7 |
| **Treatment modality** | Single modality treatment | 44 | 43.1 |
|  | Multi-modality treatment | 58 | 56.9 |

**Notes:** SD: Standard Deviations

**Table S3. Mean and Standard Deviation of MDADI**

|  | **Mean** | **SD** |
| --- | --- | --- |
| **Total questions** | 53.34 | 16.96 |
| **Global subscale** | 44.51 | 23.0 |
| **Emotional subscale** | 56.63 | 17.44 |
| **Functional subscale** | 57.69 | 17.53 |
| **Physical subscale** | 49.44 | 19.26 |
| **Composite score** | 53.29 | 15.85 |

**Notes:** MDADI: MD Anderson Dysphagia Inventory, SD: Standard Deviation
